# Comparison of Chest CT Manifestations of Coronavirus Disease 2019 (COVID-19) and Pneumonia Associated with Lymphoma

**DOI:** 10.7150/ijms.46688

**Published:** 2020-07-19

**Authors:** Shuting Wang, Yinshi Zheng, Zhaoqi Wang, Xiaoqiang Yao, Bei Dong, Huan Liu, Jinrong Qu

**Affiliations:** 1 Department of Radiology, Affiliated Cancer Hospital of Zhengzhou University, Henan Cancer Hospital, 127 Dongming Rd, Zhengzhou 450008, China.; 2 Department of Radiology, the First People's Hospital of Shangqiu, 292 South Kaixuan Rd, Shangqiu 476100, China.; 3 GE Healthcare, Shanghai 201203, China.

**Keywords:** Pneumonia, Coronavirus infection, COVID-19, Lymphoma, Computed Tomography

## Abstract

**Objective:** To retrospectively compare the clinical features and chest computed tomography (CT) characteristics of coronavirus disease 2019 (COVID-19) and pneumonia in lymphoma patients.

**Materials and Methods:** Ten lymphoma patients with pneumonia and 12 patients with COVID-19 infections were enrolled from January 15 to March 14, 2020. The clinical features were recorded. All pulmonary lesions on chest CT were assessed for location, shape, density and diffusion degree. Other typical CT features were also evaluated.

**Results:** The most commonly observed patchy lesions were ground-glass opacities (GGOs) and mixed GGOs in both groups. Regarding the diffusion degree, 82% (92/112) of the lesions in the COVID-19 group were relatively limited, while 69% (52/75) of those in the lymphoma group were diffuse (*p* < 0.001). The proportions of interlobular septal thickening, vascular thickening, pleural involvement and fibrous stripes observed in the lymphoma cases were statistically compatible with those observed in the COVID-19 cases (*p >* 0.05). Air bronchograms were observed more frequently in COVID-19 patients (45%, 50/112) than in lymphoma patients with pneumonia (5%, 4/75) (*p* < 0.001). Halo sign (6%) and reversed halo sign (1%) were observed in several COVID-19 patients but not in lymphoma-associated pneumonia patients.

**Conclusion:** Both lymphoma-associated pneumonia and COVID-19 generally manifested as patchy GGOs and mixed GGOs in more than one lobe. Compared to COVID-19, lymphoma-associated pneumonia tended to be relatively diffuse, with fewer air bronchograms, and no halo or reversed halo signs observed on chest CT.

## Introduction

Since December 2019, an ongoing outbreak of pneumonia infected with a new coronavirus has been found in Wuhan, Hubei province, and was named coronavirus disease 2019 (COVID-19) by the World Health Organization on January 30, 2020. As the infection spread, the disease spread across other provinces of China and other countries across the world 1-4. As of April 5, 2020, there are more than 1.22 million confirmed cases and more than 65000 deaths around the world.

At present, viral nucleic acid detection with real-time reverse-transcription polymerase chain reaction (RT-PCR) assay has been recommended as the reference standard to confirm the diagnosis of COVID-19 according to the latest Guideline of Diagnosis and Treatment of COVID-19 (7^th^ version) 5. Although the specificity of RT-PCR is high, the sensitivity is poor 6. Currently, chest computed tomography (CT) findings have been recommended as major evidence for a confirmed clinical diagnosis 7, 8. Previous studies reported ground-glass opacities (GGOs) and patchy consolidation in more than one lobe on COVID-19 patients' chest CT 9, 10.

During the epidemic period, tumor patients still need to receive surgery, regular radiotherapy and chemotherapy to avoid serious consequences of disease progression due to delayed treatment. In case of nosocomial infection, the patients need to be excluded from COVID-19 before systematic treatment. During the chest CT screening of tumor patients in our hospital, a large proportion of pneumonia, especially interstitial pneumonia (IP), could be found in patients with lymphoma. Previous studies have reported IP in lymphoma patients undergoing chemotherapy with or without rituximab 11, 12. More than 30% of patients with hematologic malignancies including lymphoma may develop pneumonia during the course of treatment 13. Therefore, it is crucial to differentiate COVID-19 and pneumonia associated with lymphoma, which will help blocking the epidemic transmission of COVID-19 and providing guidance for the treatment of pneumonia in lymphoma patients.

To date, the differences in the manifestations on chest CT of COVID-19 and pneumonia associated with lymphoma remain unclear. The purpose of this study was therefore to preliminarily compare the clinical features and chest CT characteristics of COVID-19 and pneumonia in lymphoma patients.

## Materials and Methods

### Clinical Features

This retrospective study was approved by the ethical committee of Henan Cancer Hospital, and informed consents were waived. Ten lymphoma patients with pneumonia and 12 COVID-19 patients confirmed by RT-PCR were enrolled from January 15, 2020 to March 14, 2020. All patients received at least one chest CT examination. The clinical symptoms and epidemiological history of each patient were recorded. The 10 lymphoma patients were excluded from having COVID-19 by hospital expert consultations based on epidemiological history, clinical symptoms, chest CT, laboratory tests, and two RT-PCR tests (sampling time at least 24 hours apart) when necessary in accordance with the 7^th^ version of the Guideline of Diagnosis and Treatment of COVID-19 5. For lymphoma patients with pneumonia, the classification of lymphoma and chemotherapy regimens were also recorded.

### CT Protocol

All chest CT examinations were performed with a multi-detector CT scanner with 64 or more channels. The patients were instructed to hold their breath during the acquisition, which included whole lung volume. The parameters were as follows: tube voltage, 120-130 kVp; automatic tube current, 60-300 mA; overall scan time, 2 s; and slice thickness for reconstruction, 1 mm.

### Qualitative CT Image Analysis

Two experienced radiologists (with 6 and 20 years of experience in chest CT respectively) reviewed the chest CT images independently through a picture archiving and communication system (PACS, Neusoft Corp., China) and reached a final agreement.

The readers identified the number of affected lobes and counted the number of lesions of each patient. Afterwards, each pulmonary lesion was assessed for location, shape, density and diffusion degree. The location of each lesion was classified as peripheral, central or peripheral and central. The peripheral area was identified as the outer one-third of the lung, while the central area was the inner two-thirds of the lung. The shape of each lesion was identified as patchy or nodular. The density of each lesion was identified as GGO, mixed GGO and consolidation or consolidation. The diffusion degree was classified as diffuse or relatively limited. Furthermore, the presence of interlobular septal thickening, vascular thickening, air bronchogram, pleural involvement, fibrous stripes, halo sign and reversed halo sign were also evaluated.

### Statistical Analysis

Continuous variables were expressed as the mean ± standard deviation and were assessed by the t-test. Categorical variables were displayed as counts and percentages, and were assessed by the chi-square test or Fisher's exact test. Statistical analyses were performed using SPSS 20.0 (IBM Corp., USA). *P* values < 0.05 were considered to be significantly different.

## Results

### Demographics

All 12 patients infected with COVID-19 had a fever, and all had a positive epidemiological history (had been to Wuhan or were in close contact with confirmed patients). One of the 10 lymphoma patients with pneumonia had a fever (*p* < 0.001), and none of the lymphoma patients had a positive epidemiological history (*p* < 0.001) (**Table 1**).

Of the lymphoma patients with pneumonia in this study, 6 (60%) cases were aged 60 or above, with 7 (70%) male patients and 3 (30%) female patients. Among the 10 lymphoma patients, 8 (80%) were diagnosed with diffuse large B-cell lymphoma, while 1 (10%) was diagnosed with ALK (+) anaplastic large cell lymphoma, and 1 (10%) was diagnosed with Hodgkin's lymphoma. Regarding the chemotherapy regimens, 8 (80%) patients received chemotherapy with rituximab, 1 (10%) patient received cyclophosphamide, doxorubicin, vincristine and prednisone (CHOP), and 1 (10%) patient received doxorubicin, vinblastine and dacarbazine (AVD) and a programmed cell death-1 (PD-1) inhibitor before the chest CT examination (**Table 2**).

### CT Image Analysis

**Figure 1:**
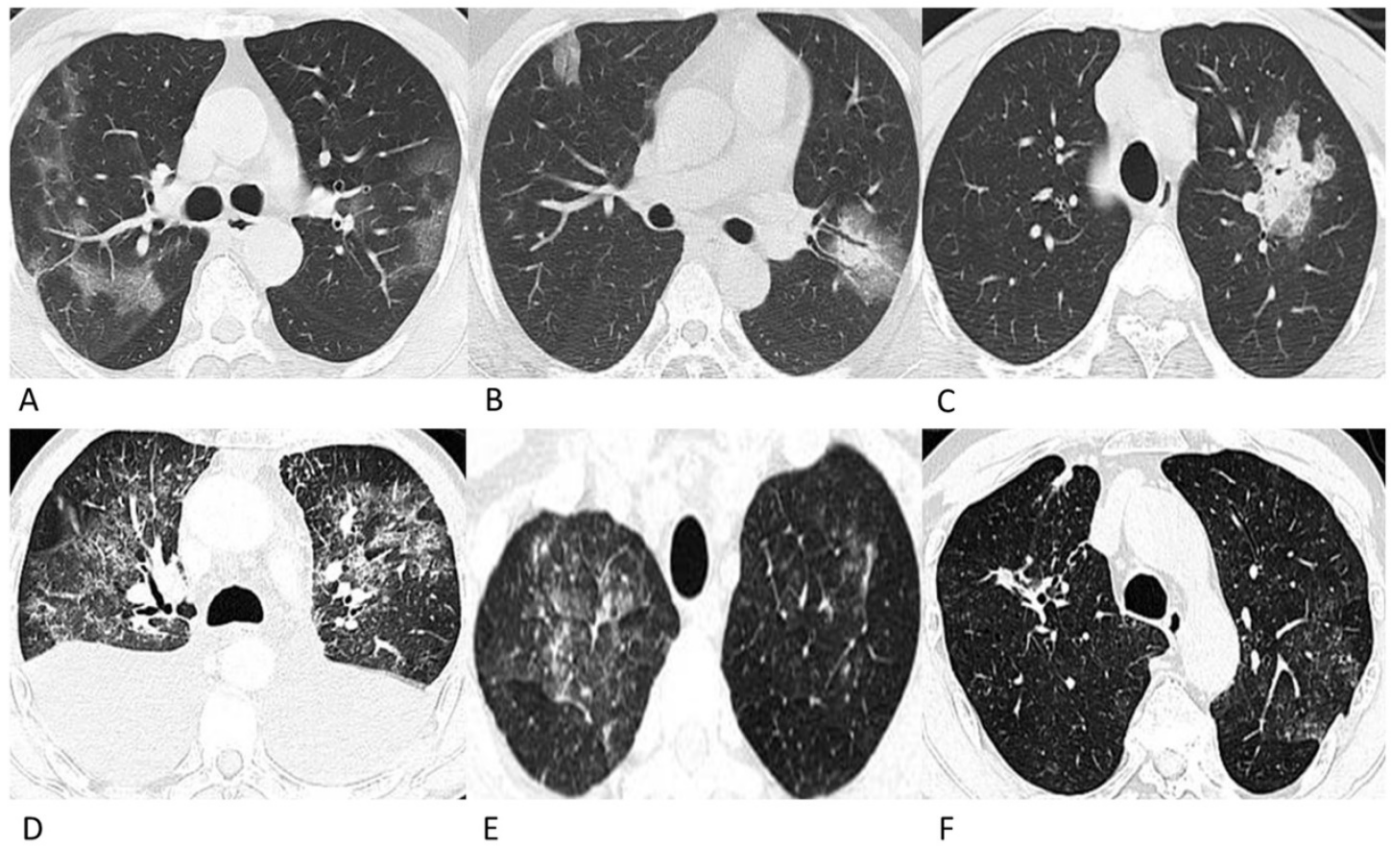
** Representative chest CT images of COVID-19 pneumonia (A-C) and lymphoma-associated pneumonia (D-F).** (A) A 62-year-old man who worked in Wuhan had a fever and chest tightness. Patchy GGOs are shown in more than one lobe. (B) A 42-year-old man, who was in close contact with a confirmed patient developed fever and cough on admission. Patchy GGOs in the peripheral right lung and mixed GGOs in the left lung are observed. Bronchograms are shown in the lesions. (C) A 28-year-old man who was in close contact with the confirmed patient in Fig. 1A developed fever. A single mixed GGO and consolidation is shown in the left lung. Focal interlobular septal thickening could be observed. (D) A 61-year-old man was diagnosed with ALK (+) anaplastic large cell lymphoma and received chemotherapy with CHOP before chest CT, and he did not have a fever or a positive epidemiological history. Diffuse patchy GGOs are shown in more than one lobe. (E) A 49-year-old man was diagnosed with diffuse large B-cell lymphoma, and received chemotherapy with rituximab before the chest CT. He had a fever during chemotherapy but had no epidemiological history of COVID-19. Diffuse patchy GGOs with several solid nodules are shown in both upper lobes. (F) A 69-year-old man was diagnosed with diffuse large B-cell lymphoma, and received chemotherapy with rituximab before the chest CT, and he did not have a fever or a positive epidemiological history. Slightly diffuse patchy GGOs are shown in the left upper lobe, and patchy consolidation was observed in the right upper lobe.

As shown in **Table 3**, no significant differences in the number of affected lobes (*p* = 0.481) or location (*p* = 0.186) were observed between the COVID-19 group and lymphoma group. The proportion of patchy lesions in the COVID-19 group and lymphoma group was 84% (94/112) and 83% (62/75), respectively (*p* = 0.843). For patchy lesions, the COVID-19 patients and lymphoma patients had the following distribution of lesion densities: GGO, 55% (52/94) versus 42% (26/62); mixed GGO and consolidation, 44% (41/94) versus 50% (31/62); and consolidation, 1% (1/94) versus 8% (5/62) (*p* = 0.036). For nodular lesions, the COVID-19 patients and lymphoma patients had the following distribution of lesion densities: GGO, 39% (7/18) versus 0% (0/13); mixed GGO and consolidation, 44% (8/18) versus 23% (3/13); and consolidation, 17% (3/18) versus 77% (10/13) (*p* = 0.002). Regarding the diffusion degree, 82% (92/112) of the COVID-19 group lesions were relatively limited, while 69% (52/75) of the lymphoma group lesions were diffuse (*p* < 0.001) (**Figure 1**).

**Figure 2:**
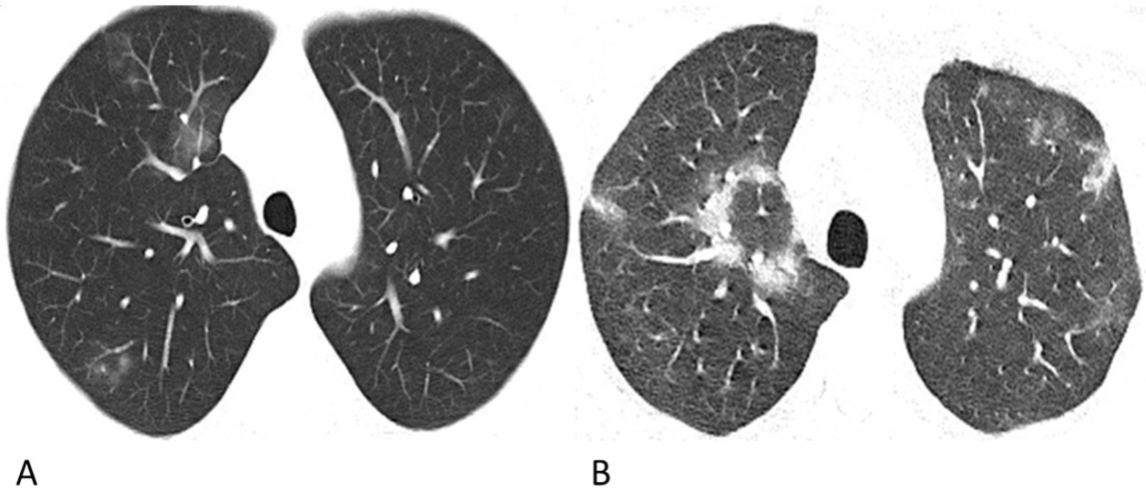
** Representative chest CT images of COVID-19 pneumonia with halo sign (A) and reversed-halo sign (B).** (A) A 28-year-old man who worked in Wuhan had a fever and cough on admission. A Patchy GGO and two mixed GGO nodules are shown in the right upper lobe, one of which with halo sign. (B) A 32-year-old man who lived in Wuhan developed fever and cough. Reversed-halo sign is shown in the right upper lobe.

**Figure 3:**
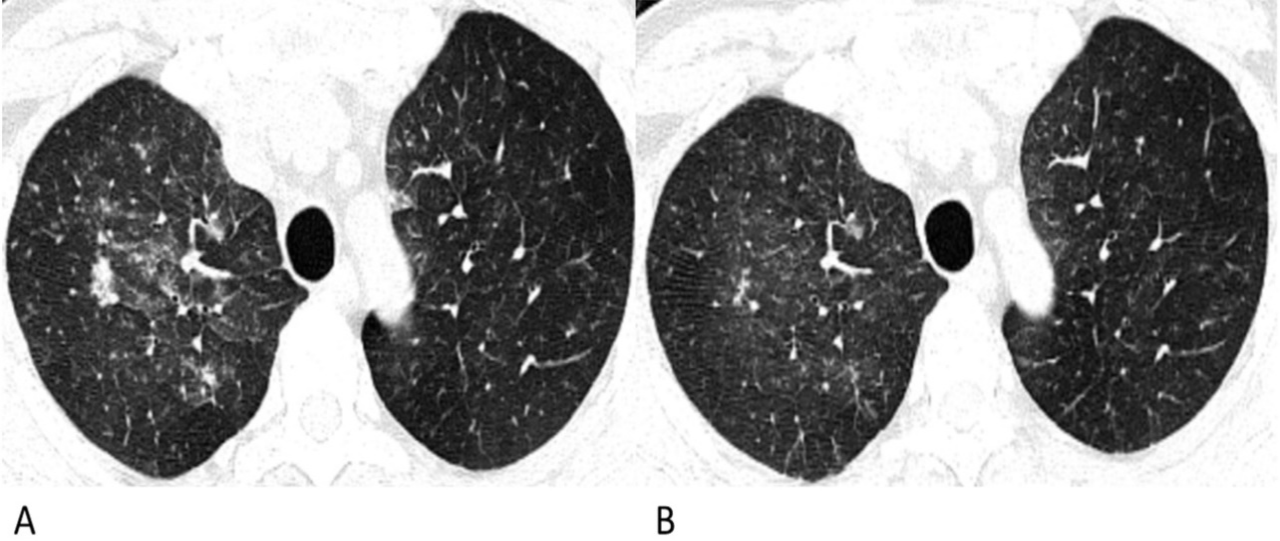
** Follow-up chest CT images of lymphoma-associated pneumonia.** A 49-year-old man was diagnosed with diffuse large B-cell lymphoma, and received chemotherapy with rituximab before the chest CT. He had a fever during the chemotherapy, but had no epidemiological history of COVID-19. (**A**) On the initial chest CT, diffuse patchy GGOs with several solid nodules are shown in both upper lobes. (**B**) After antibiotic and symptomatic treatment, the lesions in the follow-up CT are diminished, and the density decrease.

The proportions of CT features such as interlobular septal thickening, vascular thickening, pleural involvement and fibrous stripes observed in the lymphoma cases were statistically compatible with those observed in the COVID-19 cases (*p >* 0.05). Air bronchograms were seen much more frequently in COVID-19 patients (45%, 50/112) than in lymphoma patients with pneumonia (5%, 4/75) (*p* < 0.001). Among these lesions, the halo sign (6%) and reversed halo sign (1%) were observed in several COVID-19 patients (**Figure 2**), while no halo sign or reversed halo sign was observed in lymphoma patients with pneumonia. The representative follow-up chest CT images of lymphoma patients were shown in **Figure 3**.

## Discussion

Our study showed that both lymphoma-associated pneumonia and COVID-19 generally manifested as patchy GGOs and mixed GGOs in more than one lobe on chest CT. Lymphoma-associated pneumonia tended to be relatively diffuse, with fewer air bronchograms than COVID-19 pneumonia. Pneumonia, especially IP, is a substantial comorbidity in lymphoma patients. The incidence of IP was 2.9%-3.8% in patients with lymphoma 12, 14. Chemotherapy with rituximab was identified as an independent risk factor for IP 14, 15. PD-1 inhibitors were also reported to be related to pneumonia in advanced cancer patients including lymphoma patients 16. In the group of lymphoma patients with pneumonia in this study, 8 (80%) patients received chemotherapy with rituximab, 1 (10%) patient received CHOP, and 1 (10%) patient received chemotherapy with AVD and a PD-1 inhibitor before the chest CT scan. The development of pneumonia in lymphoma patients may be associated with the application of rituximab and cytotoxic drugs (such as cyclophosphamide, doxorubicin, epirubicin, and PD-1) and mixed infections due to agranulocytosis. During the epidemic period of COVID-19, differentiating pneumonia associated with lymphoma from COVID-19 can help stop the spread of the infectious disease and provide guidance for the treatment of pneumonia in lymphoma patients. The clinical symptoms, epidemiological history and chest CT findings might play a critical role in obtaining a prompt diagnosis.

In the present study, all 12 patients with COVID-19 had a fever and a positive epidemiological history, while for the lymphoma patients with pneumonia, only 1 (10%) patient had a fever, and none of the lymphoma patients had a positive epidemiological history (*p* < 0.001). The clinical features and epidemiological history play an important role in screening patients infected with COVID-19. According to the 7^th^ version of the Guideline of Diagnosis and Treatment of COVID-19 5, patients with a positive epidemiological history and 2 clinical symptoms or patients with no epidemiological history but with 3 clinical symptoms as well as positive chest CT features are defined as suspected patients who need to receive RT-PCR or virus gene sequencing of their respiratory or blood specimens.

Recent studies have shown that chest CT findings appear earlier than clinical manifestations; therefore, chest CT has been recommended as major evidence for the screening, clinical diagnosis and evaluation of lesion progression and therapeutic effects 17, 18. The chest CT characteristics of COVID-19 pneumonia in this study were generally consistent with those in previous studies 18-20, with patchy (84%) GGO and consolidated lesions in more than one lobe (83%) in the peripheral lung (53%). Moreover, vascular thickening (54%), air bronchogram (45%), pleural involvement (25%), fibrous stripes (20%), and interlobular septum thickening (8%) were recorded in this group of confirmed cases (**Figure 1**). It is worth mentioning that among these lesions, halo sign (6%) and reversed halo sign (1%) were observed in several patients, which have also been reported in a few recent studies 21, 22 (**Figure 2**). The halo sign is defined as GGO surrounding a nodule or consolidation and has been initially described in hemorrhagic nodules, which can be typically observed in invasive fungal infections. This sign could also be seen in cryptococcosis and lung neoplasms 23. The reversed halo sign refers to a focal rounded area of GGO surrounded by a ring of consolidation, which is typically observed in granulomatous diseases and organizing pneumonia 24. Moreover, when the reversed halo sign is peripheral and with internal reticulation, this suggests pulmonary infarction, and computed tomography angiography is necessary to evaluate the possible pulmonary embolism 25. Recently, both organizing pneumonia 26 and pulmonary infarction 27 have been observed in COVID-19 patients, which indicates that organizing pneumonia and pulmonary infarction are potential mechanisms of lung injury in COVID-19. Extensive pathological significance of the two CT manifestations observed in COVID-19 patients warrants further study. Despite the similarities in CT manifestations, the proportion of the relatively limited lesion margin was 82% in our cases of COVID-19 patients, while only 30% lesions were well-defined in a previous study 10; this may be due to the differences in the evaluation criteria, which refers to the diffusion degree of the lesion in this study.

The CT manifestations of pneumonia in the lymphoma patients in this study showed several similarities to those of COVID-19 patients. The proportion of patients with multi-lobar infections was 100% in lymphoma patients with pneumonia and 83% in COVID-19 patients (*p* = 0.481). Lesions in the peripheral area of the lung presented in 39% of the lymphoma patients with pneumonia and in 53% of COVID-19 patients. For patchy lesions, the most commonly observed densities were GGO and mixed GGO and consolidation in both lymphoma patients with pneumonia (50% and 42%) and COVID-19 patients (44% and 55%). Moreover, the proportions of CT features such as interlobular septal thickening, vascular thickening, pleural involvement and fibrous stripes observed in the lymphoma cases were compatible with those observed in the COVID-19 cases.

However, for lymphoma patients with pneumonia, solid nodules occupied 77% in nodular lesions, while for COVID-19 patients, GGO and mixed GGO nodules occupied 83% (*p* = 0.002). Notably, compared with the lesions of COVID-19 patients which were relatively limited or confined, the lesions of lymphoma patients were much more diffuse in the lung, like the “diffuse mist” (*p* < 0.001) (Fig. 1). This may be a critical characteristic in identifying pneumonia associated with lymphoma from that associated with COVID-19. Furthermore, air bronchograms were seen less frequently in lymphoma patients with pneumonia than in COVID-19 patients (5% vs. 45%,* p* < 0.001). Additionally, no halo sign or reversed halo sign was observed in lymphoma patients with pneumonia. After antibiotic and symptomatic treatment, the patchy lesions on the follow-up CT of the lymphoma patient decreased (Fig. 3); therefore, the patient was able to tolerate continuous chemotherapy.

There are several limitations in the present study. First, considering the importance of urgent reporting, the number of enrolled patients was small, and it was unable to analyze the CT manifestations of pneumonia induced by different pathogens in lymphoma patients. Further work needs to be performed to increase the number of patients, for an intensive evaluation of the CT manifestations of lymphoma-associated pneumonia and COVID-19 pneumonia. Second, some of the patients had only one initial chest CT. In subsequent research, follow-up CT examinations are needed to enable dynamic evaluations of the progression of the lesions and the therapeutic effect. Third, in the present study, some lymphoma patients with pneumonia did not receive RT-PCR. This is because they were excluded from suspected COVID-19 cases by hospital expert consultations in accordance with the 7^th^ version of the Guideline of Diagnosis and Treatment of COVID-19. The subsequent relief of the symptoms and CT manifestations also supported the diagnosis.

In conclusion, both lymphoma-associated pneumonia and COVID-19 generally manifested as patchy GGOs and mixed GGOs in more than one lobe, with interlobular septal thickening, vascular thickening, pleural involvement and fibrous stripes in the lung. Compared to COVID-19, lymphoma-associated pneumonia tended to be relatively diffuse, with fewer air bronchograms, no halo or reversed halo sign observed in the chest CT. During the epidemic period of COVID-19, for lymphoma patients with pneumonia on chest CT, the clinical symptoms, epidemiological history and laboratory tests should also be taken into consideration. When necessary, RT-PCR needs to be performed for differentiation. On the other hand, follow-up CT is necessary to monitor the progression of the disease. Radiologists and clinicians should be familiar with the typical CT characteristics of COVID-19 and pneumonia associated with lymphoma, as these signs are valuable for blocking the epidemic transmission of COVID-19 and providing guidance for the treatment of pneumonia in lymphoma patients.

## Figures and Tables

**Table 1:** Demographic of the COVID-19 patients and lymphoma patients

	COVID-19	Lymphoma	P
Age	43 ± 17	59 ± 12	0.025*
M/F	8/4	7/3	1.000
Fever	12/0	1/9	< 0.001*
Epidemiological history			< 0.001*
Yes	9	0	
No	3	10	

*P values less than 0.05 were considered statistically significant.

**Table 2:** Supplementary demographics of the lymphoma patients

	Number	(%)
**Type**		
Diffuse Large B-cell Lymphoma	8	80
ALK (+) anaplastic large cell lymphoma	1	10
Hodgkin's lymphoma	1	10
**Chemotherapy regimens**		
Rituximab involved	8	80
CHOP	1	10
AVD and PD-1 inhibitor	1	10

AVD: doxorubicin, vinblastine and dacarbazine. CHOP: cyclophosphamide, doxorubicin, vincristine, and prednisone. PD-1: programmed cell death-1.

**Table 3:** Chest CT characteristics of COVID-19 patients and pneumonia in lymphoma patients

	COVID-19 N (%)	Lymphoma N (%)	P
**Number of affected lobes**			0.481
One lobe	2 (17)	0 (0)	
More than one lobe	10 (83)	10 (100)	
Total number of lesions	112	75	
**Location**			0.186
Peripheral	59 (53)	29 (39)	
Central	36 (32)	31 (41)	
Peripheral and Central	17 (15)	15 (20)	
**Patchy lesions**	94	62	0.036**
GGO	52 (55)	26 (42)	
Mixed GGO and consolidation	41 (44)	31 (50)	
Consolidation	1 (1)	5 (8)	
**Nodular lesions**	18	13	0.002*
GGO	7 (39)	0 (0)	
Mixed GGO and consolidation	8 (44)	3 (23)	
Consolidation	3 (17)	10 (77)	
**Diffusion degree**			< 0.001*
Diffuse	20 (18)	52 (69)	
Relatively limited	92 (82)	23 (31)	
**Interlobular septal thickening**	9 (8)	12 (16)	0.103
Vascular thickening	61 (54)	30 (40)	0.073
Air bronchogram	50 (45)	4 (5)	< 0.001*
Pleural involvement	28 (25)	14 (19)	0.373
Fibrous stripes	22 (20)	17 (23)	0.714
Halo sign	7 (6)	0 (0)	0.043*
Reversed halo sign	1 (1)	0 (0)	1.000

*P values less than 0.05 were considered statistically significant; **P values of pairwise comparisons were 0.024, 0.242 and 0.09 respectively, with no significant difference after Bonferroni correction.

## Abbreviations

COVID-19: coronavirus disease 2019
RT-PCR: reverse-transcription polymerase chain reaction
CT: computed tomography
GGO: ground-glass opacity
IP: interstitial pneumonia
CHOP: cyclophosphamide, doxorubicin, vincristine and prednisone
AVD: doxorubicin, vinblastine and dacarbazine
PD-1: programmed cell death-1
